# Pathogen-specific deep sequence-coupled biopanning: A method for surveying human antibody responses

**DOI:** 10.1371/journal.pone.0171511

**Published:** 2017-02-02

**Authors:** Kathryn M. Frietze, Juan M. Pascale, Brechla Moreno, Bryce Chackerian, David S. Peabody

**Affiliations:** 1 Department of Molecular Genetics and Microbiology, University of New Mexico School of Medicine, MSC08 4660, 1 University of New Mexico, Albuquerque, NM, United States of America; 2 Gorgas Memorial Institute for Health Studies, Ave. Justo Arosemena y Calle 35, Panamá, Panamá; Icahn School of Medicine at Mount Sinai, UNITED STATES

## Abstract

Identifying the targets of antibody responses during infection is important for designing vaccines, developing diagnostic and prognostic tools, and understanding pathogenesis. We developed a novel deep sequence-coupled biopanning approach capable of identifying the protein epitopes of antibodies present in human polyclonal serum. Here, we report the adaptation of this approach for the identification of pathogen-specific epitopes recognized by antibodies elicited during acute infection. As a proof-of-principle, we applied this approach to assessing antibodies to Dengue virus (DENV). Using a panel of sera from patients with acute secondary DENV infection, we panned a DENV antigen fragment library displayed on the surface of bacteriophage MS2 virus-like particles and characterized the population of affinity-selected peptide epitopes by deep sequence analysis. Although there was considerable variation in the responses of individuals, we found several epitopes within the Envelope glycoprotein and Non-Structural Protein 1 that were commonly enriched. This report establishes a novel approach for characterizing pathogen-specific antibody responses in human sera, and has future utility in identifying novel diagnostic and vaccine targets.

## Introduction

Knowing the targets of the antibodies that are elicited in response to natural infection is important for developing vaccines and new diagnostic tests. However, our ability to comprehensively and quantitatively characterize the epitopes targeted by individual antibodies in a polyclonal population is limited. Recent efforts to couple deep-sequencing technologies with phage display-based biopanning provides an alternative and complementary strategy for characterizing epitopes targeted in complex polyclonal serum [1–6]. We recently described a strategy that uses peptide libraries displayed on the bacteriophage MS2 virus-like particle (MS2-VLP) affinity selection platform and deep sequence analysis to identify epitopes targeted in serum from ovarian cancer patients [5]. Here, we report the adaptation of this method to the characterization of linear, pathogen-associated B-cell epitopes targeted during acute infection with a pathogen. As a proof-of-prinicple we chose to focus on dengue virus (DENV).

DENV comprises 4 serotypes (DENV-1,-2,-3,-4) with considerable genetic variation within types. A first infection with DENV (primary infection) generates a long-lasting protective immune response to the infecting DENV serotype and some degree of cross-protection against other DENV serotypes [7]. However, heterospecific protection is thought to wane after 6 months, after which individuals are susceptible to secondary DENV infection. Secondary infection is a risk factor for severe dengue (SD), including dengue hemorrhagic fever (DHF) and dengue shock syndrome (DSS). Although the specific reasons for this phenomenon are not well understood, the most common mediator is thought to be non-neutralizing antibodies that enhance DENV infection and is referred to as antibody-dependent enhancement (ADE) of infection [8]. Secondary infection results in antibody responses able to neutralize all four DENV serotypes [7]. Although active surveillance for DENV infection and seroconversion in cohorts indicates that tertiary and quaternary infections of DENV occur, these infections are almost always clinically inapparent, suggesting that the broadly neutralizing antibody response generated after secondary infection is sufficient to protect against clinical dengue disease [9]. Such epitopes could, in principle, provide the basis for vaccines that broadly protect against diverse dengue serotypes.

Our understanding of the complex antibody response to infectious diseases has been limited by a lack of methods with which to comprehensively characterize the specific epitopes targeted during natural infection. Pepscan technology allows the identification of linear epitopes but is limited by cost of peptide synthesis and the sensitivity of the assay [10, 11]. More recent efforts have utilized deep sequencing technologies coupled with traditional phage display to try to comprehensively characterize antibody responses to infectious diseases [1].

Here, we describe an approach for mapping the antibody repertoire against an infectious disease in humans that utilizes a pathogen-specific antigen fragment library displayed on bacteriophage MS2-VLPs in combination with deep sequence-coupled biopanning. As a proof-of-principle, we chose to focus on DENV because of its relatively simple proteome and used available human serum samples from patients with acute DENV secondary infection. Using this approach, we generated a detailed map of the linear epitopes targeted by antibody responses to secondary DENV infection in humans and present an approach that can be readily applied to other pathogens of interest.

## Materials and Methods

### Patient serum samples

Patient serum samples were obtained from DENV-infected patients seven days post-onset of fever. Samples were identified as primary or secondary infection as follows: primary infection as IgM positive/IgG negative, and secondary infection as IgM and IgG positive. Serum samples were tested for DENV IgM by Panbio Dengue IgM Capture ELISA and DENV IgG Capture ELISA (Alere, Inc.) and manufacturer’s algorithm for identifying primary vs. secondary infection was used. Samples were de-identified to UNM researchers and consisted of primary DENV infection samples (n = 31) and secondary DENV infection samples (n = 30). One secondary DENV infection sample was chosen randomly for an initial pilot experiment with two rounds of biopanning. Nine additional secondary DENV infection samples were chosen (in addition to the pilot sample) for further studies with one round of biopanning. Institutional Review Board approval was granted by both institutions involved in this study (Bioethics Research Committee of the Gorgas Memorial Institute for Health Studies and the UNMHSC/School of Medicine IRB Committee).

### Generation of bacteriophage MS2 VLP DENV-3 antigen fragment library

We produce libraries of peptides on MS2 VLPs by site–directed mutagenesis of MS2 coat protein in pDSP62 [12] after the method of Kunkel [13]. In this case we made a library containing all possible overlapping 10-mers from the dengue polyprotein sequence. The nearly 4,000 primers needed to fully represent the dengue polyprotein sequence were produced by LC Sciences using a chip-based parallel synthesis method. They had the following general structure: 5’-GTTCTCGTCGACAATGGC[XXX]_10_GGCGACGTGACTGTCGCC-3’, where XXX corresponds to *E*. *coli* optimized codons corresponding to every possible 10-mer from the DENV-3 polyprotein. The sequences flanking the dengue inserts anneal to the MS2 coat protein sequences at a site corresponding to the coat protein AB-loop. Since the chip-based synthesis method normally yields only about 10–20 pmol of DNA, the primer mixture was polymerase chain reaction (PCR) amplified using Priamp1 (5’-GGCAACTTTACTCAGTTCGTTCTCGTCGACAATGGC-3’) and Priamp2 (5’-GTTAGCGAAGTTGCTTGGGGCGACAGTCACGTCGCC-3’), which results in 102-bp products. Ten percent of the product of the first PCR reaction was then used as template for a second PCR reaction using only Priamp2 primer (0.5nmol). This causes linear amplification of the antisense strand, which is then used as the primer for site-directed mutagenesis [13] on single-stranded dUTP-substituted pDSP62 plasmid, which expresses coat protein from the phage T7 promoter. The resulting covalently-closed circular DNA was then introduced into *E*. *coli* (strain 10G) for amplification of the plasmid library, which was then introduced by electroporation into *E*. *coli* strain C41(DE3) where the VLP library was synthesized (MS2-DENV3 VLP AFL). The resulting complex mixture of VLPs was purified by methods described previously [12].

### Deep sequence-coupled biopanning

IgG was isolated from 20μL of patient serum using Dynabeads Protein G (Invitrogen) following the manufacturer’s instructions. The concentration of IgG was estimated using absorbance at 280nm using a Nanodrop. IgG (4μg) was mixed with 10μg of DENV-3 antigen fragment library displayed on MS2-VLPs in a total volume of 100μL with phosphate-buffered saline (PBS). This was incubated for 1 hour at room temperature with shaking. VLP/antibody complexes were then pulled down using 20μL Dynabeads Protein G with incubation at room temperature for 40 minutes. Complexes were then washed 6 times in 0.5% Tween PBS (PBS-T) and 3 times in PBS (200μL volumes), transferring to fresh tubes after wash 1, wash 4, and wash 7. VLPs were then eluted for 5 min with 50μL 0.1M glycine pH 2.7 and neutralized immediately with 5μL 1M Tris pH 9.0. Eluted VLPs were then used as template in RT-PCR using the following conditions: 4.0μL E2 primer (5’-TCAGCGGTGGCAGCAGCCAA-3’) with 1.0μL dNTP mix (Invitrogen) and 8μL eluted VLPs heated to 65°C for 5 min and quick chilled on ice. Next, 4.0μL of 5X First Strand Buffer (Invitrogen) and 0.1M dithiothreitol were added and heated to 37°C for 2 min. Finally, 1μL Maloney Murine Leukemia Virus Reverse Transcriptase (Invitrogen) was added and reaction was incubated at 37°C for 50 minutes followed by 15 min at 70°C. Reverse transcriptase-reaction (2μL) was used in PCR with 5μL 10X PCR buffer, 2μL MgSO_4_, 1μL dNTP mix (Invitrogen), 62up primer (5’-CTATGCAGGGGTTGTTGAAG-3’), E3.2 primer (5’-CGGGCTTTGTTAGCAGCCGG-3’), and 0.2μL Platinum HiFi Taq polymerase (Invitrogen) in a 50μL total reaction volume. PCR product was confirmed by agarose gel electrophoresis and then purified with Qiaquick PCR Purification kit (Qiagen) using manufacturer’s instructions. Purified PCR product was digested with *Bam*HI and *Sal*I-HF (New England Biolabs) and then ligated with appropriately digested pDSP62(am) low valency plasmid [12]. After ethanol precipitation of the ligation reaction, electrocompetent 10G *E*. *coli* cells were transformed, recovered in 100mL LB at 37°C for 1 hour, kanamycin antibiotic was added to 50μg/mL and cultures were incubated overnight at 37°C with shaking. Plasmid DNA was isolated with Qiaprep Maxiprep Kit (Qiagen) following the manufacturer’s instructions. C41(DE3)/pNMsupA cells [12] were then electroporated with 250ng of plasmid DNA, diluted into 50mL LB medium and shaken at 37°C. After 1 hour 50μg/mL kanamycin and 25μg/mL chloramphenicol were added, and growth was continued until the culture reached OD 600nm of 0.6. Synthesis of coat protein was induced by addition of Isopropyl β-D-1-thriogalactopyranoside (IPTG) to a final concentration of 0.4mM, and after an additional 3 hrs at 37°C, cells were harvested by centrifugation and the pellets frozen at -80°C. VLP isolation was carried out as previously described [12]. When additional rounds of selection were required, repetition of the procedures described above.

Upon completion of the last selection round, plasmid DNA was purified, digested with *Kpn*I and then used as template for 15 cycles of PCR with Ion Torrent barcoded primers. Note that pDSP62 contains a unique *Kpn*I site that is destroyed when a foreign peptide is inserted, so digestion with the enzyme prevents amplification of any residual contaminating pDSP62. PCR products were then run on a 1.2% agarose gel in Tris-Borate-EDTA buffer (45 mM Tris-Borate, 1 mM EDTA), excised with a scalpel, and extracted using Qiaquick Gel Extraction kit (Qiagen) following the manufacturer’s instructions. This purified PCR product was then subjected to Ion Torrent Deep Sequencing. Raw data was processed with custom MATLAB scripts as previously described [5]. Sequences that passed quality control standards were then used to identify the unique peptides, determine the abundance of each, and rank them according to their abundance (% total population of quality controlled sequences). The starting DENV-3 antigen fragment plasmid library was also subjected to Ion Torrent Deep Sequencing and used to determine fold-enrichment of each peptide (% population sequences for patient biopanning / % population sequences of starting library). For peptides identified in patient biopanning samples but not present in the starting library, we assumed an abundance of 1 read in order to calculate fold enrichment.

### Structures and alignments

Alignments of peptides were performed with NCBI BLAST against the reference sequence for Dengue virus 3 polyprotein (accession #: YP_001621843.1). Structures for DENV envelope dimer (PDB#: 1UZG), trimer (PBD#: 4GTO), and NS1 (PDB#: 4O6B) were generated in Cn3D [14].

### Synthetic peptide ELISA

Streptavidin (0.01μg/μL in PBS) was added to Immunolon 96-well ELISA plates in 200μL volumes and incubated overnight at 4°C. Plates were washed 3 times in PBS and succinimidyl 6-((beta-maleimidopropionamido)hexanoate)) (SMPH) heterobifunctional crosslinker was added (0.02μg/μL) in 200μL volumes and incubated at room temperature for 2 hours. Plates were washed 3 times in PBS and custom synthetic peptides containing a C-term cysteine (Genscript) were added at 0.02μg/μL in 200μL volumes and incubated at room temperature for 2 hours. Plates were washed 3 times and then blocked overnight at 4°C with 0.5% dry milk/PBS. The following day, blocking solution was removed and 200μL of 1:100 diluted (in 0.5% dry milk/PBS) patient serum samples were added. This was incubated at room temperature for 2 hours, washed with PBS-T, and secondary horseradish peroxidase-conjugated donkey anti-human IgG (Jackson ImmunoResearch) was added at 1:5000 dilution (in 0.5% dry milk/PBS) in 200μL volumes. After incubation for 1 hour at room temperature, plates were washed with PBS-T followed by a wash with PBS, and 200μL of SureBlue Reserve TMB (KPL, Inc.) developing reagent was added. Plates were incubated at RT with shaking until sufficiently developed (~5 min), and read at 630nm.

### Statistical analysis

Statistical analyses of data were carried out using Prism 4 for Macintosh. For ELISA on patient serum samples, Mann-Whitney U test was used. P values of <0.05 were considered significant.

## Results

### Construction of an MS2-VLP DENV-3 antigen fragment library

**Fig 1:**
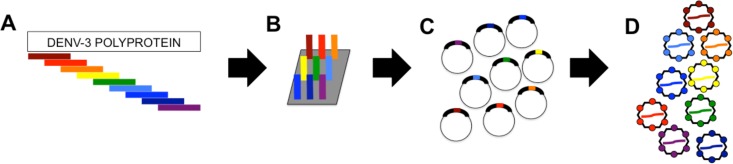
Generation of MS2-VLP DENV-3 antigen fragment library. (A) The DENV-3 polyprotein was used to identify all possible 10 amino acid peptides, overlapping by 9 amino acids. (B) *E*. *coli* codon optimized coding sequences were synthesized by a massively parallel microchip-based synthesis-on-chip technique (LC Sciences), and (C) then used to generate a corresponding plasmid library in the pDSP62 vector. (D) The library was then expressed as VLPs in *E*. *coli* and used in subsequent deep sequence-coupled biopanning experiments.

**Fig 2:**
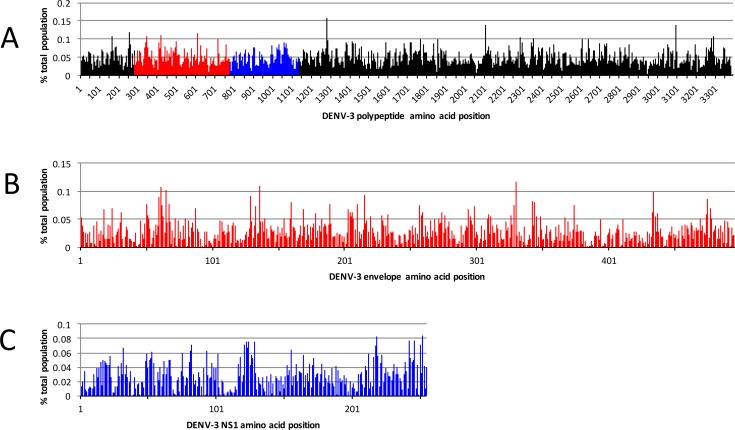
DENV-3 plasmid starting library. DENV-3 antigen fragment plasmid library was assessed by Ion Torrent Deep Sequencing to determine coverage at each amino acid position. (A) Deduced peptide sequences were aligned to the DENV-3 polyprotein with NCBI BLAST and used to determine the coverage at each amino acid position. Each peak represents the % total population of reads representing peptides that align starting at that amino acid position. The red and blue regions represents the E protein and NS1 protein, respectively. (B and C) The E protein and NS1 protein coverage is enlarged from (A) in order to show the coverage of the plasmid library for these regions.

We previously reported the utility of the MS2-VLP platform for identifying the epitopes recognized by monoclonal antibodies and polyclonal serum [5, 6, 12, 15, 16]. This work required the generation of highly diverse, random peptide libraries displayed on the surface of the MS2-VLPs followed by several rounds of biopanning to enrich for binders. In order to adapt the MS2-VLP affinity selection platform specifically to the identification of linear epitopes recognized by sera of DENV-infected patients, we generated an antigen fragment library (AFL) of the DENV-3 polyprotein (Fig 1). We chose DENV-3 as the basis for the AFL because our patient serum samples were obtained during a period in which DENV-3 and DENV-1 circulated in Panama. The library was constructed by synthesizing overlapping 30 nucleotide oligonucleotides corresponding to the DENV-3 genome using a massively parallel microchip-based synthesis method. After amplification by PCR, the oligonucleotides were then used to construct a library of VLP expression plasmids encoding every possible 10-mer DENV-3 peptide. Ion Torrent Deep Sequencing of the plasmid library indicated that it largely covered the entire DENV-3 polyprotein sequence (Fig 2). The library was then expressed as VLPs in *E*. *coli* and used in subsequent deep sequence-coupled biopanning experiments.

### Deep sequence-coupled biopanning of DENV secondary infection patient serum

**Fig 3:**
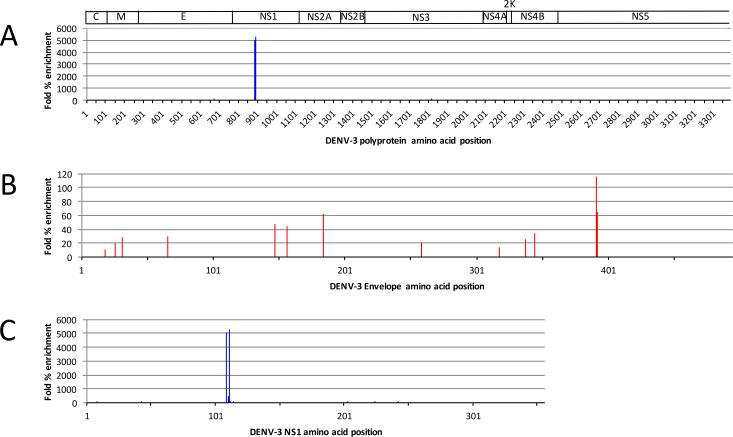
Deep sequencing results from two rounds of deep sequence-coupled iterative biopanning with serum sample 1536. (A) Fold % enrichment of each peptide is presented for the entire DENV-3 polyprotein. (B and C) E and NS1 protein regions are shown enlarged from (A). Note the change in y-axis scale.

Deep sequence-coupled biopanning was performed using a subset of available human serum samples isolated from 10 patients with secondary DENV infection. A pilot experiment involving two rounds of affinity-selection with patient sample 1536 showed that the selected population was highly enriched for just a handful of library members (Fig 3), represented primarily by an overlapping epitope in the NS1 protein (Fig 3C), hereafter referred to as LKYSWKTWGKAK. Although enriched to lesser extents, several other peptides were also identified, including some corresponding to a region at the junction of domain III and the stem region of the E-protein (Fig 3B), hereafter called KKGSSIGKMFE.

**Fig 4:**
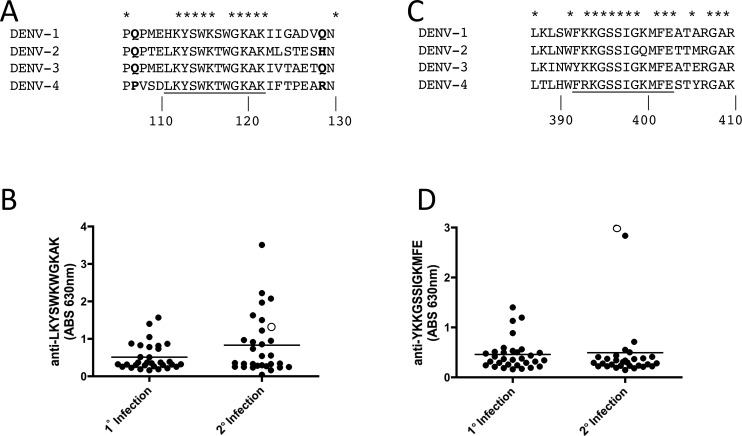
NS1 and E enriched epitopes identified by deep sequence-coupled biopanning with patient serum after two rounds of biopanning. NS1 conservation (A) is shown for the highly enriched overlapping peptides from patient 1536 after two rounds of biopanning. Bold letters indicate the flanking amino acids that are solved in the crystal structure (see Fig 5F for structure). E conservation (C) is shown for the most enriched overlapping peptides from patient 1536 after two rounds of biopanning. ELISAs against synthetic peptides representing the NS1 epitope (B) and the E epitope (D) are shown. Open circles represent the data point for patient 1536 (the sample used to identify the epitopes). Primary serum samples (n = 31) and secondary serum samples (n = 30) isolated from patients 7 days post onset of fever were used.

**Fig 5:**
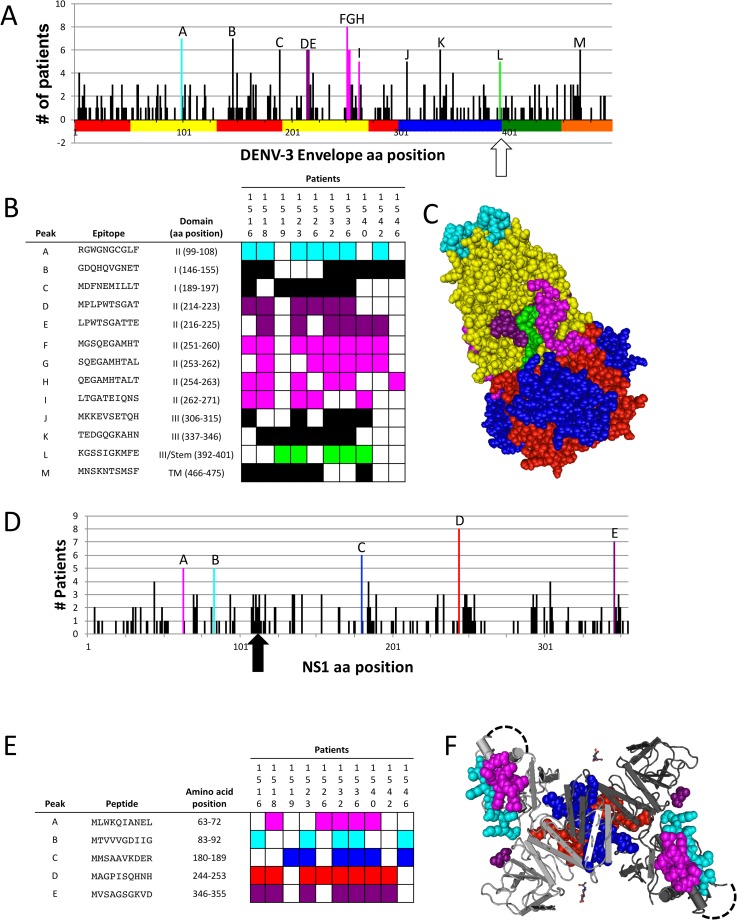
Commonly targeted E- and NS1 peptides. (A and D) The number of patients with a peptide enriched (10-fold or greater enrichment above starting library) is indicated at each amino acid position for E (A) and NS1 (D). Bars are indicated with letters are peptides enriched in 5 or more patients to highlight the commonly targeted epitopes (A and D). Individual E (B) and NS1 (E) peptides targeted by at least 5 patients are shown and colors correspond to the trimer (fusion) conformation of E (C) or NS1 (F). Dotted black lines in (F) represent the putative disordered region in which the LKYSWKTWGKAK epitope is located and is shown in (D) with an arrow. Colors of residues in (A) correspond to the colors of residues in the structure shown in (C). The junction of domain III and the stem region of E is indicated by an open arrow.

Interestingly, both the NS1 LKYSWKTWGKAK and E-protein KKGSSIGKMFE epitopes are highly conserved across DENV serotypes (Fig 4A and 4C). Inspection of the known structures of these proteins [17–20] shows LKYSWKTWGKAK corresponds to a disordered loop on NS1 (Fig 5F) and KKGSSIGKMFE is located at the junction of domain III (DIII) and the stem region of E-protein (Fig 5C). Structural studies of the various conformational intermediates of DENV E indicate that the KKGSSIGKMFE epitope is buried in the pre-fusion conformation of E, but is exposed during conformational changes that accompany the fusion process [20].

### Antibody responses to LKYSWKTWGKAK and KKGSSIGKMFE in DENV patients

In order to determine if antibody responses to these two epitopes were commonly observed, we used a panel of serum from acute primary (n = 31) and secondary DENV-infected individuals (n = 30) and performed ELISA against synthetic peptides corresponding to both epitopes (Fig 4B and 4D). Interestingly, sera from patients with secondary infections tended to show higher antibody reactivity to LKYSWKTWGK than sera from patients with primary DENV infection, although the difference was not statistically significant. Very few patients reacted with YKKGSSIGKMFE (Fig 4B and 4D). The selecting patient serum sample (Fig 4B and 4D, open circles) reacted strongly with both YKKGSSIGKMFE and LKYSWKTGKAK peptides by ELISA, confirming that our deep sequence-coupled biopanning method indeed identified linear epitopes targeted by IgG from patient 1536.

### Identification of commonly targeted epitopes using a single round of deep sequence-coupled biopanning

Our initial experiment using two rounds of deep sequence-coupled biopanning resulted in a population highly enriched for a single NS1 epitope (LKYSWKTGKAK) with much lower enrichment for E peptides. Concerned that two selection rounds might oversimplify the selectant population, we sequenced the products of a single round of biopanning. This time, selection was conducted using sera from all 10 patients, and data analysis focused on peptides that were commonly selected. A selected epitope was defined as a sequence that was represented at a frequency at least 10-fold above the starting plasmid library (S1 Fig).

### Commonly targeted DENV E and NS1 epitopes

Antibodies to E and NS1 have been implicated in pathogenesis, protection, neutralization, and enhancement of infection. Thus, we focused our efforts on identifying shared enriched E (Fig 5A–5C) and NS1 (Fig 5D–5F) peptide epitopes. Peptides enriched at least 10-fold by at least 5 patients were identified and are presented in Fig 5. Most shared E-protein peptides are found in domain II, including several that are represented by overlapping peptides (Fig 5A–5C, peaks D/E, peaks F/G/H/I). One of these domain II peptides corresponds to the DENV fusion-loop (Fig 5A–5C, peak A). The others flank the KKGSSIGKMFE peptide in the fusion trimer conformation of E-protein, for which 5 patient samples, including patient 1536 (from the pilot biopanning experiment) showed enrichment (Fig 5A–5C, Peak L).

Five NS1 peptides were enriched by 5 or more patients. Two pairs of peptides (Peaks A/B, Peaks C/D) are adjacent to one another in the NS1 dimer (Fig 5D–5F). Peaks A and B are located on one of the NS1 “arms”, while Peaks C and D cluster at the interface of the NS1 dimer. Although the LKYSWKTWGKAK epitope, originally identified after two selection rounds with patient 1536 sera, didn’t meet the 5 or more patient threshold, antibodies targeting a cluster of peptides overlapping the epitope were identified in several patients (Fig 5D, arrow). One explanation for this is that the LKYSWKTWGKAK epitope is commonly targeted, but individual patients recognize distinctly different peptides in this region.

## Discussion

Previous efforts to characterize the antibody response to linear epitopes for DENV infection have been limited to versions of the so-called Pepscan technology to identify only E-associated antibody responses in serum samples from individuals with primary infection (i.e. a monotypic antibody responses) for serotypes 2 and 3 [10, 11]. These assays have limited sensitivity for detection of individual epitopes that elicit relatively low antibody titers and is hindered by the high cost of peptide synthesis. In contrast, our method readily generates a complete representation of the entire proteome and takes advantage of deep sequence analysis to identify epitopes that provoke even relatively weak antibody responses. Our library is generated in *E*. *coli* with the highest cost being the initial synthesis of the oligonucleotide library, which is considerably lower than synthesis of peptide libraries. Interestingly, only 3 of the epitopes we identified were also identified by previous Pepscan efforts (Peaks E, G, and M)[10, 11]. This suggests that our approach may be more sensitive than Pepscan for identifying antibody responses during infection, and/or that secondary infection patient serum has unique repertoires of antibodies that are not represented during primary infection. Interestingly, we identified antibody responses in 7 patients corresponding to the DENV E fusion loop (Fig 5A–5C, Peak A) and 5 patients to the stem region (Fig 5A–5C, Peak L), both of which are highly conserved among flaviviruses. These epitopes are buried in the E dimer until they are exposed during fusion of the viral and host membranes.

The NS1 epitope we identified by deep sequence-coupled biopanning with patient 1536 serum sample has been identified before in other contexts. The LKYSWKTWGKAK contains the previously described LX-1 epitope YSWKTWG [21]. Our data suggests that secondary infection patient sera have higher reactivity to the LKYSWKTWGKAK peptide than primary infection patients, although the difference did not reach statistical significance with this set of patient samples (Fig 4B). Another study identified key residues *KXWG* that are recognized by polyclonal serum from mice immunized with NS1 and trigger antibody-dependent complement-mediated cytotoxicity [22]. These antibodies cross-react with the LYRIC (lysine-rich CEACAM1 co-isolated) protein (also known as metadherin, 3D3, or astrocyte-elevated gene-1 (AEG-1)) in endothelial cells suggesting a role for anti-NS1 antibodies that cross-react with host proteins in some of the hemorrhage-related sequelae of DENV infection [22]. However, a recent report showed that NS1-immune polyclonal mouse serum or monoclonal antibodies to NS1, and immunization with NS1 protected mice against DENV-2 in a lethal challenge model [23], suggesting a role for anti-NS1 antibodies in protection from disease. One of our NS1 epitopes (Fig 5D–5F, Peak D) was recently identified as a target of human anti-NS1 monoclonal antibodies [24]. In that report, nine human monoclonal antibodies to NS1 from patients secondarily infected with DENV-2 bound to a common epitope located between amino acids 221–266. We found that sera from 8/10 patients in our panel significantly enriched for peptides in this region (Fig 5D–5F, Peak D, aa 244–253). As efforts to generate NS1-targeted vaccines to DENV are underway, it will be important to further characterize the specificity of anti-NS1 antibodies in patients in order to understand their role in pathogenesis and protection.

Here, we describe an approach for targeted deep sequence-coupled biopanning with patient serum which, when coupled with appropriate bioinformatics analysis, can inform efforts for diagnostic tools and vaccines. The strength of our deep sequence-coupled biopanning approach for characterizing commonly targeted epitopes in infection lies in its scalability, speed, and ability to broadly characterize the entire pathogen-targeted antibody repertoire at once. From the initial design of the MS2-VLP peptide library construction to the identification of epitopes of interest, our deep sequence-coupled biopanning approach can be carried out in as little as 1 week. Subsequent engineering of those epitopes onto MS2-VLPs for immunization studies in mice can be carried out in less than a week. Although we focus this report on NS1 and E epitopes, we were able to identify shared epitopes targeted against other DENV proteins as well. DENV encodes a relatively small polyprotein, but we have created random peptide libraries with complexities >10^10^, meaning that we could use this approach for applications requiring much larger numbers of peptides. Our targeted deep sequence-coupled biopanning approach is technically limited by the size of peptides that can be displayed in the MS2 AB loop, library censorship against some hydrophobic peptides, and an inability to identify conformational epitopes. Although approaches similar to ours have been described [1, 3, 4], our approach is unique in that our platform (MS2 VLPs) displaying an identified peptide can be directly used as an immunogen if desired and is capable of eliciting strong antibody responses *in vivo* [12, 25]. We foresee various iterations of our deep sequence-coupled biopanning approach for characterizing antibody responses in polyclonal serum samples, including the identification of diagnostic targets for new and emerging infections, characterization of antibody responses in humans or animals upon infection with various pathogens, or antibody-mediated disease states.

## Conclusions

In this work, we engineered MS2 VLPs to display an antigen fragment library of the DENV-3 proteome. This antigen fragment library was then used for deep sequence-coupled biopanning with DENV-infected patient serum to investigate the antibody responses of secondary infected patients. We identified common B-cell epitopes among patients, including several interesting epitopes on the DENV proteins E and NS1. This proof-of-principle study demonstrates that this approach can be easily adapted to investigate antibody specificity for other infectious diseases, aiding in the rapid identification of diagnostic tools and vaccines.

## Supporting Information

S1 Fig E-associated peptides identified for each DENV secondary infection patients. Deep sequence-coupled biopanning results are plotted showing peptides (black lines) that were enriched 10-fold or more for each patient. Shading indicates the associated domain of E.
(PDF)

## References

[1] Xu GJ, Kula T, Xu Q, Li MZ, Vernon SD, Ndung’u T, et al Comprehensive serological profiling of human populations using a synthetic human virome. Science. 2015;348(6239). 10.1126/science.aaa0698 PMC4844011 26045439

[2] Larman HB, Laserson U, Querol L, Verhaeghen K, Solimini NL, Xu GJ, et al PhIP-Seq characterization of autoantibodies from patients with multiple sclerosis, type 1 diabetes and rheumatoid arthritis. J Autoimmun. 2013;43:1–9. Epub 2013/03/19. PubMed Central PMCID: PMCPmc3677742. 10.1016/j.jaut.2013.01.013 23497938 PMC3677742

[3] Larman HB, Zhao Z, Laserson U, Li MZ, Ciccia A, Gakidis MA, et al Autoantigen discovery with a synthetic human peptidome. Nat Biotechnol. 2011;29(6):535–41. Epub 2011/05/24. 10.1038/nbt.1856 21602805 PMC4169279

[4] Ryvkin A, Ashkenazy H, Smelyanski L, Kaplan G, Penn O, Weiss-Ottolenghi Y, et al Deep Panning: steps towards probing the IgOme. PLoS One. 2012;7(8):e41469 Epub 2012/08/08. PubMed Central PMCID: PMCPmc3409857. 10.1371/journal.pone.0041469 22870226 PMC3409857

[5] Frietze KM, Roden RB, Lee JH, Shi Y, Peabody DS, Chackerian B. Identification of Anti-CA125 Antibody Responses in Ovarian Cancer Patients by a Novel Deep Sequence-Coupled Biopanning Platform. Cancer Immunol Res. 2016;4(2):157–64. PubMed Central PMCID: PMCPMC4740227. 10.1158/2326-6066.CIR-15-0165 26589767 PMC4740227

[6] Crossey E, Frietze K, Narum DL, Peabody DS, Chackerian B. Identification of an Immunogenic Mimic of a Conserved Epitope on the Plasmodium falciparum Blood Stage Antigen AMA1 Using Virus-Like Particle (VLP) Peptide Display. PLoS One. 2015;10(7):e0132560 PubMed Central PMCID: PMCPMC4493041. 10.1371/journal.pone.0132560 26147502 PMC4493041

[7] Wahala WM, Silva AM. The human antibody response to dengue virus infection. Viruses. 2011;3(12):2374–95. PubMed Central PMCID: PMCPMC3280510. 10.3390/v3122374 22355444 PMC3280510

[8] Halstead SB. Antibodies determine virulence in dengue. Ann N Y Acad Sci. 2009;1171 Suppl 1:E48–56. 19751402 10.1111/j.1749-6632.2009.05052.x

[9] Montoya M, Gresh L, Mercado JC, Williams KL, Vargas MJ, Gutierrez G, et al Symptomatic versus inapparent outcome in repeat dengue virus infections is influenced by the time interval between infections and study year. PLoS Negl Trop Dis. 2013;7(8):e2357 Epub 2013/08/21. PubMed Central PMCID: PMCPmc3738476. 10.1371/journal.pntd.0002357 23951377 PMC3738476

[10] Innis BL, Thirawuth V, Hemachudha C. Identification of continuous epitopes of the envelope glycoprotein of dengue type 2 virus. Am J Trop Med Hyg. 1989;40(6):676–87. 2472749 10.4269/ajtmh.1989.40.676

[11] da Silva AN, Nascimento EJ, Cordeiro MT, Gil LH, Abath FG, Montenegro SM, et al Identification of continuous human B-cell epitopes in the envelope glycoprotein of dengue virus type 3 (DENV-3). PLoS One. 2009;4(10):e7425 PubMed Central PMCID: PMCPMC2760205. 10.1371/journal.pone.0007425 19826631 PMC2760205

[12] Chackerian B, Caldeira Jdo C, Peabody J, Peabody DS. Peptide epitope identification by affinity selection on bacteriophage MS2 virus-like particles. J Mol Biol. 2011;409(2):225–37. Epub 2011/04/20. 10.1016/j.jmb.2011.03.072 21501621 PMC3095728

[13] Kunkel TA. Rapid and efficient site-specific mutagenesis without phenotypic selection. Proc Natl Acad Sci U S A. 1985;82(2):488–92. PubMed Central PMCID: PMCPMC397064. 3881765 10.1073/pnas.82.2.488 PMC397064

[14] Wang Y, Geer LY, Chappey C, Kans JA, Bryant SH. Cn3D: sequence and structure views for Entrez. Trends Biochem Sci. 2000;25(6):300–2. 10838572 10.1016/s0968-0004(00)01561-9

[15] O'Rourke JP, Daly SM, Triplett KD, Peabody D, Chackerian B, Hall PR. Development of a mimotope vaccine targeting the Staphylococcus aureus quorum sensing pathway. PLoS One. 2014;9(11):e111198 Epub 2014/11/08. PubMed Central PMCID: PMC4224382. 10.1371/journal.pone.0111198 25379726 PMC4224382

[16] Ord RL, Caldeira JC, Rodriguez M, Noe A, Chackerian B, Peabody DS, et al A malaria vaccine candidate based on an epitope of the Plasmodium falciparum RH5 protein. Malar J. 2014;13:326 Epub 2014/08/20. PubMed Central PMCID: PMC4152569. 10.1186/1475-2875-13-326 25135070 PMC4152569

[17] Klein DE, Choi JL, Harrison SC. Structure of a dengue virus envelope protein late-stage fusion intermediate. J Virol. 2013;87(4):2287–93. Epub 2012/12/14. PubMed Central PMCID: PMCPmc3571469. 10.1128/JVI.02957-12 23236058 PMC3571469

[18] Zhang X, Ge P, Yu X, Brannan JM, Bi G, Zhang Q, et al Cryo-EM structure of the mature dengue virus at 3.5-A resolution. Nat Struct Mol Biol. 2013;20(1):105–10. Epub 2012/12/18. PubMed Central PMCID: PMCPmc3593067. 10.1038/nsmb.2463 23241927 PMC3593067

[19] Akey DL, Brown WC, Dutta S, Konwerski J, Jose J, Jurkiw TJ, et al Flavivirus NS1 structures reveal surfaces for associations with membranes and the immune system. Science. 2014;343(6173):881–5. PubMed Central PMCID: PMCPMC4263348. 10.1126/science.1247749 24505133 PMC4263348

[20] Modis Y, Ogata S, Clements D, Harrison SC. Structure of the dengue virus envelope protein after membrane fusion. Nature. 2004;427(6972):313–9. 10.1038/nature02165 14737159

[21] Falconar AK. Antibody responses are generated to immunodominant ELK/KLE-type motifs on the nonstructural-1 glycoprotein during live dengue virus infections in mice and humans: implications for diagnosis, pathogenesis, and vaccine design. Clin Vaccine Immunol. 2007;14(5):493–504. PubMed Central PMCID: PMCPMC1865631. 10.1128/CVI.00371-06 17329445 PMC1865631

[22] Liu IJ, Chiu CY, Chen YC, Wu HC. Molecular mimicry of human endothelial cell antigen by autoantibodies to nonstructural protein 1 of dengue virus. J Biol Chem. 2011;286(11):9726–36. PubMed Central PMCID: PMCPMC3058979. 10.1074/jbc.M110.170993 21233208 PMC3058979

[23] Beatty PR, Puerta-Guardo H, Killingbeck SS, Glasner DR, Hopkins K, Harris E. Dengue virus NS1 triggers endothelial permeability and vascular leak that is prevented by NS1 vaccination. Sci Transl Med. 2015;7(304):304ra141 10.1126/scitranslmed.aaa3787 26355030

[24] Omokoko MD, Pambudi S, Phanthanawiboon S, Masrinoul P, Setthapramote C, Sasaki T, et al A highly conserved region between amino acids 221 and 266 of dengue virus non-structural protein 1 is a major epitope region in infected patients. Am J Trop Med Hyg. 2014;91(1):146–55. PubMed Central PMCID: PMCPMC4080554. 10.4269/ajtmh.13-0624 24778195 PMC4080554

[25] Peabody DS, Manifold-Wheeler B, Medford A, Jordan SK, do Carmo Caldeira J, Chackerian B. Immunogenic display of diverse peptides on virus-like particles of RNA phage MS2. J Mol Biol. 2008;380(1):252–63. PubMed Central PMCID: PMCPMC2481506. 10.1016/j.jmb.2008.04.049 18508079 PMC2481506

