# Supplementary material for: Pathogen-specific deep sequence-coupled biopanning: A method for surveying human antibody responses
Source: PLoS One. 2017 Feb 2;12(2):e0171511. doi: 10.1371/journal.pone.0171511 (PMC5289605; doi:10.1371/journal.pone.0171511)
Supplement: S1 Fig — Deep sequence-coupled biopanning results are plotted showing peptides (black lines) that were enriched 10-fold or more for each patient. Shading indicates the associated domain of E. (PDF) [file pone.0171511.s001.pdf]

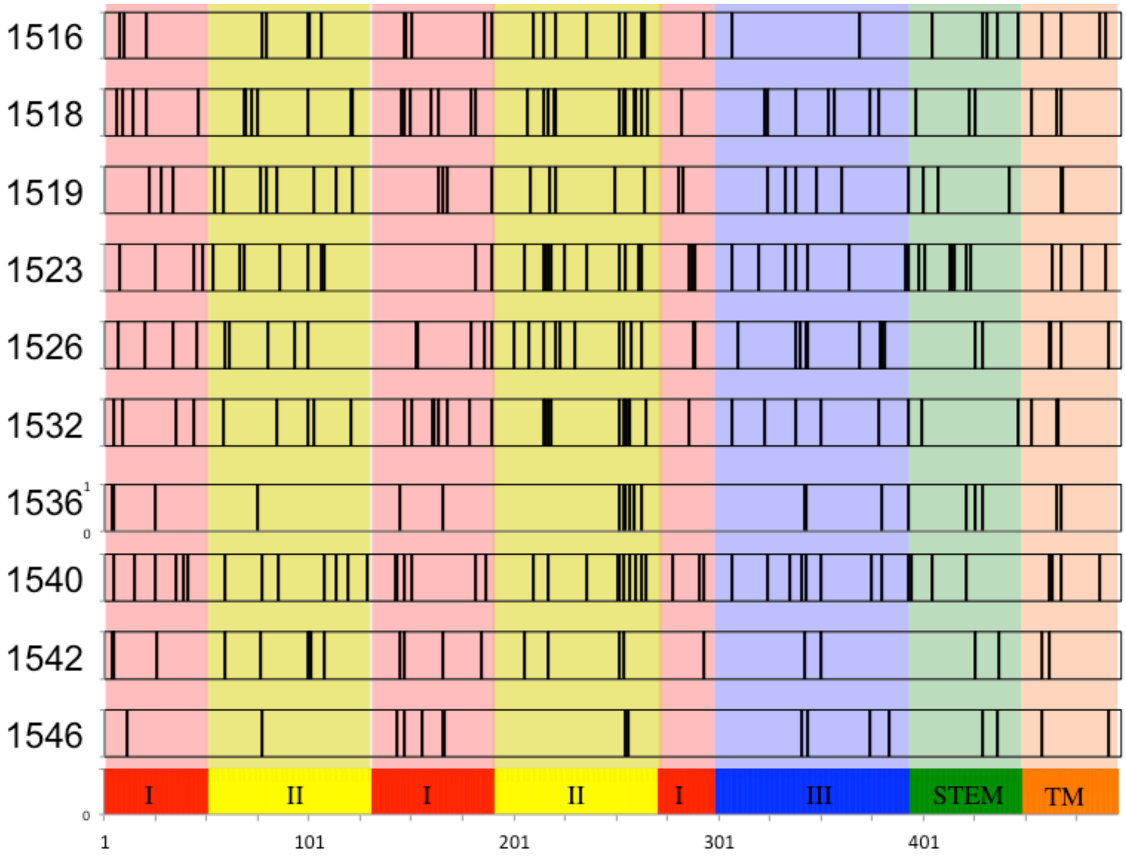

S1 Figure. E-associated peptides identified for each DENV secondary infection patients. Deep sequence-coupled biopanning results are plotted showing peptides (black lines) that were enriched 10-fold or more for each patient. Shading indicates the associated domain of E.
